# Differences in Body Fat Distribution Play a Role in the Lower Levels of Elevated Fasting Glucose amongst Ghanaian Migrant Women Compared to Men

**DOI:** 10.1371/journal.pone.0066516

**Published:** 2013-06-19

**Authors:** Mary Nicolaou, Anton E. Kunst, Wim B. Busschers, Irene G. van Valkengoed, Henriette Dijkshoorn, Linda Boateng, Lizzy M. Brewster, Marieke B. Snijder, Karien Stronks, Charles Agyemang

**Affiliations:** 1 Department of Public Health, Academic Medical Centre, University of Amsterdam, Amsterdam, The Netherlands; 2 Department of Epidemiology, Documentation and Health Promotion, Municipal Health Service of Amsterdam, Amsterdam, The Netherlands; 3 Departments of Internal and Vascular Medicine, Academic Medical Centre, University of Amsterdam, Amsterdam, The Netherlands; CUNY, United States of America

## Abstract

**Background:**

Despite higher levels of obesity, West African migrant women appear to have lower rates of type 2 diabetes than their male counterparts. We investigated the role of body fat distribution in these differences.

**Methods:**

Cross-sectional study of Ghanaian migrants (97 men, 115 women) aged 18–60 years in Amsterdam, the Netherlands. Weight, height, waist and hip circumferences were measured. Logistic regression was used to explore the association of BMI, waist and hip measurements with elevated fasting glucose (glucose≥5.6 mmol/L). Linear regression was used to study the association of the same parameters with fasting glucose.

**Results:**

Mean BMI, waist and hip circumferences were higher in women than men while the prevalence of elevated fasting glucose was higher in men than in women, 33% versus 19%. With adjustment for age only, men were non-significantly more likely than women to have an elevated fasting glucose, odds ratio (OR) 1.81, 95% CI: 0.95, 3.46. With correction for BMI, the higher odds among men increased and were statistically significant (OR 2.84, 95% CI: 1.32, 6.10), but with consideration of body fat distribution (by adding both hip and waist in the analysis) differences were no longer significant (OR 1.56 95% CI: 0.66, 3.68). Analysis with fasting glucose as continuous outcome measure showed somewhat similar results.

**Conclusion:**

Compared to men, the lower rates of elevated fasting glucose observed among Ghanaian women may be partly due to a more favorable body fat distribution, characterized by both hip and waist measurements.

## Introduction

West African migrants form a significant proportion of the population in many industrialized countries. In 2008, the International Organization for Migration estimated that there are approximately 800,000 registered West African migrants in the main European receiving countries [1]. In 2009, 1.5 million African migrants resided in the United States, with approximately one third from West African countries including Ghana [2].

African migrants to Western Europe often have high levels of overweight and obesity and related chronic diseases [3]. Available evidence indicates that it is women who are affected most by obesity [4], [5], suggesting that women are likely to have the highest risk of obesity related chronic disease such as type 2 diabetes mellitus (DM). Unfortunately, information on the health of this migrant group is limited [3], but there are indications that, contrary to expectations, DM is more common among men than women. For example, the Health Survey for England (2004) indicated higher prevalence of doctor diagnosed DM in African-origin men (4.3%) than women (2.0%), despite much higher rates of obesity (BMI≥30) in women (38%) versus men (17%) [5]. A Canadian study by Creatore et al showed that sub-Saharan African women had lower DM prevalence rates than their male counterparts [6].

The high levels of obesity and related chronic diseases are not only a problem among migrants; there are indications that obesity and chronic disease is increasing in West African countries themselves as a result of economic development and the nutrition transition [7]. Studies in sub-Saharan Africa have also noted differences between men and women in obesity and DM. Alberts et al reported that South African rural women were much more likely to be overweight and obese than men, but levels of DM were similar (at approximately 8%) [8]. Amoah found lower rates of DM and impaired fasting glucose among women than men despite the fact that women had higher BMI and waist circumference (WC) [9]. Finally, in a review of DM prevalence in West Africa, Abubakhari et al observed lower levels of DM among women despite higher obesity levels [10].

Sex differences in DM have been reported in other populations. A Canadian study by Lipscombe et al found that men were more likely than women to have DM [11]. Meisinger et al’s study also showed higher incidence of DM among German men than women [12]. Additionally, a recent Scottish study found that men were more likely to be diagnosed with DM at lower BMI levels than women [13], which is consistent with earlier findings in the Finnmark study [14] and a US study [15]. The latter three studies postulate that sex differences in the relationship BMI and DM are likely to be due to differences in body fat distribution.

It is universally accepted that generalized obesity (measured by BMI) or abdominal obesity (measured by waist circumference) confer risk for DM, and as a result these two measures are used in identifying risk populations [16]. However, it is also clear that fat distribution plays a role in predicting DM [17], [18]. Specifically, intra-abdominal fat (visceral fat) rather than subcutaneous fat confers a higher risk for DM, due to an increased free fatty acid release into the portal system [19]. Peripheral fat seems to be associated with a decreased risk, when taking abdominal fat into account [20], [21], also in multi-ethnic populations [22], [23]. It has been postulated that this effect is due to differences in lipolytic activity between subcutaneous abdominal fat and subcutaneous thigh fat. The latter is more likely to take up free fatty acid from the circulation, resulting in wider hips or thighs, and thereby protecting other organs against free fatty acid exposure and ectopic fat storage, which is related to insulin resistance and DM [24].

Sex differences in body composition, specifically body fat distribution (visceral versus subcutaneous fat) and muscle mass, have been recognized [25], [26]. However, we found no studies that explored sex differences in the association between fasting glucose and body fat distribution in African migrant populations, and the ways in which these potentially affect sex differences in DM risk. It may be that the sex differences in the rates of DM observed in African populations are, in part, due to sex differences in body fat distribution. Therefore, in this study we aimed to explore this hypothesis in a population of first generation Ghanaian migrants to the Netherlands.

## Methods

### Ethics statement

The Medical Ethical Committee of the Academic Medical Centre, University of Amsterdam approved the study protocols (MEC 10/054). Participants completed a written informed consent form prior to the physical examination.

### Study design, population and recruitment

The GHAIA study (acronym for: **GHa**naians **I**n **A**msterdam) was a cross-sectional study with the aim of assessing the cardiovascular risk profile among this largest sub-Saharan African population in the Netherlands. Currently, there are approximately 20,000 officially recorded Ghanaians in the Netherlands [27]. Ghanaian ethnicity is officially defined on the basis of country of birth: a person is “Ghanaian” if he/she is born in Ghana or if he/she is born in the Netherlands and their parents were both born in Ghana. Most Ghanaians in the Netherlands came in the 1970s and 1980s due to economic downturn in Ghana [28] and many live in South East Amsterdam [29]. There are also a large number of undocumented (i.e. without legal resident status) Ghanaians, with estimations that this group consists of up to 20,000 individuals. In 2000, for example, about 40,000 Ghanaians registered to vote at the Ghanaian embassy for the presidential elections in Ghana in 2000 [28].

The GHAIA study was based on a sample of 18–60 year old, non-institutionalized people in Amsterdam, the Netherlands during April till October 2010. The methods are described elsewhere [30], but briefly, we aimed to include approximately 200 participants, drawn from six Ghanaian churches and an organization representing the Ghanaian Muslim community in southeast Amsterdam. This approach was used, as we did not wish to exclude undocumented Ghanaians. Our experience from previous work [4] showed that involvement of the community leaders enhances the study participation rate among these communities.

Based on the advice of key-figures within the community, we visited each of the churches involved in the study and presented the aims and objectives of the study. In each church, we took the names and contact details of those present at the time. From these lists (a total of 560), a random sample of 299 (53% women) people was drawn. Potential participants were approached for a structured interview, based on a questionnaire and conducted by bi-lingual interviewers of Ghanaian ethnicity. A total of 221 (100 men and 121 women) participated in the interview (overall response rate 73.9%). Those who responded to the interview were subsequently invited for a physical examination, to which 212 (70.9%) participated. Approximately 25% of our sample consisted of undocumented persons.

### Measurements

Information on demographics (highest education level), health and health behavior (smoking, and physical activity) was obtained during the participant's interview. Physical activity was measured using the validated “Short Questionnaire to Assess Health Enhancing Physical Activity” (SQUASH) [31]. Participants were classified as meeting the norm for physical activity if they reported engaging in moderate intensity physical activity for at least 30 minutes on 5 days of the week,

Fasting venous blood was taken for the measurement of glucose levels (HK/Glucose-6-P dehydrogenase test; Roche Diagnostics, In). Trained research assistants, using a standardized protocol, conducted anthropometric measures. Weight was measured in light clothing to the nearest 0.1 kg using an electronic scale (SECA erecta 844). Height was measured without shoes with a wall-mounted roll-up measuring tape (SECA 206) to the nearest 0.1 cm. Waist circumference was measured at midway between the lower rib margin and the iliac crest and hip circumference was measured at the widest point, over the trochanters. Both circumferences were measured in standing participants after normal expiration. Body mass index (BMI) was calculated as weight (kg) divided by height (m) squared. Abdominal obesity was defined as waist circumference ≥102 and ≥88 cm and elevated waist-to-hip ratio ≥0.95 ≥0.85 for men and women, respectively [32].

In this study we focus on elevated fasting glucose and diabetes. Elevated fasting glucose can be considered to be an intermediate stage of abnormal glucose regulation which can eventually lead to diabetes [33]. We defined elevated fasting glucose as fasting plasma glucose levels greater than 5.5 mmol/L and extended the definition to include participants with fasting glucose > 6.9 mmol/L (persons that would normally be categorized as having DM, n = 11). Furthermore, we included participants that reported receiving treatment for diabetes (n = 13, all of these participants had a fasting glucose above 5.5 mmol/L in our study). In order to simplify the description of the results in this paper we refer to the combined dichotomous outcome measure as elevated fasting glucose (EFG).

### Analysis

We tested differences in the characteristics between men and women using the Mann-Whitney test for continuous variables and the Fisher’s exact test for dichotomous variables.

Logistic regression analyses were performed to study the association of sex (main effect) with the presence of EFG. In the logistic models we adjusted for anthropometric measures as continuous variables. Associations are expressed as odds ratios (ORs) with their 95% confidence intervals. We tested for interaction between sex and all other co-variables (age, BMI, waist and hip circumferences) but found no statistically significant interactions. The final model (including all anthropometric parameters) had a concordance index (L-index) of 0.742 indicating relatively good fit.

We also repeated the analysis using linear regression with a continuous measure of fasting plasma glucose as dependent variable. As the errors in the analysis with fasting glucose did not have a normal distribution we explored different transformations using Box-Cox theory. This suggested that the optimal transformation (in order to achieve normally distributed errors) for fasting glucose was Glucose^−1.5^. The analysis applying this transformation showed similar results as the model based on untransformed fasting glucose (results available on request). For the sake of simplicity in interpretation of the analysis, we chose not to transform fasting glucose in presenting the results of the linear regression in this paper.

In both logistic and linear regression models, we tested the influence of potential confounders, smoking, education level, physical activity and length of residence in the Netherlands by adding each individually to the final model.

The three anthropometric variables were highly correlated with each other, (see table S1 for Spearman’s correlation coefficients). Thus we tested for multicollinearity by calculating the tolerance statistic and the variance inflation factors (VIF). Analysis was performed using IBM SPSS 19 and R.

## Results

**Table 1** shows characteristics of the study participants. All participants were first generation migrants, residence duration in the Netherlands varied from less than 1 year to 30 years with women having longer mean residence duration than men. Women had higher mean BMI, WC and HC than men and significantly more women were overweight or obese and, based on sex-specific cut-offs, had abdominal obesity and an elevated waist-to-hip ratio. Average fasting glucose levels did not differ between men and women, but men were more often categorized as having an EFG than women. Smoking levels were low in this population (1%) and did not differ between the sexes, as did either physical activity level or highest attained education level. We performed additional tests to explore differences in baseline characteristics between documented and undocumented participants and found no differences between the groups (data not shown).

**Table 1:** Characteristics of study participants.

Variable	Men (n = 97)	Women (n = 115)	P-value
Median age, yrs. (IQR)	47.0 (13)	45.0 (11)	p = 0.363
Mean height, cm (SD)	170.0 (0.06)	159.0 (0.06)	p = 0.580
Mean weight, kg (SD)	78.1 (11.3)	79.3 (14.8)	p = 0.142
Mean waist circumference, cm (SD)	89.5 (10.3)	93.8 (13.2)	p = 0.546
Mean hip circumference, cm (SD)	92.2 (7.7)	104.1 (11.9)	p = 0.428
Mean BMI, kg/m^2^ (SD)	27.0 (3.4)	31.3 (5.5)	p = 0.448
Overweight, n (%) *BMI = 25.0*–*29.9*	45 (46.4)	43 (37.4)	p = 0.185
Obesity, n (%) *BMI≥30.0*	22 (22.7)	61 (53.0)	p = 0.0001
Abdominal obesity^1^, n (%)	10 (10.3)	76 (66.1)	p = 0.0001
Median waist-to-hip ratio (IQR)	0.96 (0.10)	0.90 (0.08)	p = 0.0001
Elevated waist-to-hip ratio^2^ (%)	58.8	76.5	p = 0.006
Median fasting plasma glucose, mmol/L (IQR)	5.2 (1.0)	5.1 (0.7)	p = 0.247
Elevated fasting glucose, n (%)	32 (33.0)	23 (20.0)	p = 0.041
Mean residence duration in the Netherlands, yrs (SD)	13.9 (7.6)	16.3 (6.5)	p = 0.032
Current smoker (%)	1.0	1.0	p = 0.270
Physical activity norm^3^, yes (%)	42.3	45.2	p = 0.606
Tertiary education level (%)	33.0	25.0	p = 0.001

1 Abdominal obesity defined on the basis of waist circumference: in men ≥102 cm in women ≥88 cm. ^2^ Elevated waist-to-hip ratio defined in men ≥0.95 and women ≥0.85.

3 Physical activity norm is engaging in moderate intensity physical activity for at least 30 minutes on 5 days of the week.

**Table 2** shows sex differences in EFG. Compared to women, men had greater age-adjusted odds of EFG although this difference was not statistically significant. However, when adjusting for BMI, sex differences were considerably larger and statistically significant, with men having almost 3 times greater odds of EFG. Sex differences persisted with correction for either WC or HC, but the differences between men and women were reduced and no longer significant when the three anthropometric variables were added concurrently in the analysis, or when the waist-to-hip ratio was added instead of WC or HC individually. In the models where WC and HC were added concurrently, their relative contributions to EFG were opposing, i.e. WC was positively associated with EFG whereas HC was negatively associated with EFG.

**Table 2:** Odds Ratio of elevated fasting glucose (EFG) for men compared to women, adjusted for anthropometric variables.

	Sex^1^	BMI	Waist circumference	Hip circumference
	O.R. (95% CI)	O.R. (95% CI)	O.R. (95% CI)	O.R. (95% CI)
Adjusted for age	1.81 (0.95, 3.46)			
Adjusted for age + BMI	2.84 (1.32, 6.10)	1.09 (1.02, 1.17)		
Adjusted for age + waist + hip	1.47 (0.64, 3.38)		1.11 (1.05,1.17)	0.93 (0.88, 0.99)
Adjusted for age + BMI waist + hip	1.56 (0.66, 3.68)	1.10 (0.95, 1.26)	1.08 (1.02, 1.15)	0.92 (0.86, 0.98)

Data are Odd Ratio (95% Confidence Interval). ^1^ Women are the reference category.

**Table 3** shows the association of fasting glucose levels with sex corrected for the measured anthropometric variables. This analysis revealed no statistically significant sex differences in fasting glucose.i.e. there was no evidence that the association between obesity and diabetes differed on the basis of sex. However, results of further analyses correspond to those for EFG: the contribution of BMI to fasting glucose was no longer significant when both WC and HC were added to the model and WC and HC had significant opposite associations with fasting glucose levels. We found no evidence that multicollinearity disturbs the models in which multiple anthropometric variables were added: the tolerance statistic was >0.1 and the VIF was <5 for all variables.

**Table 3:** Age-adjusted associations of sex with fasting plasma glucose levels, adjusted for anthropometric variables.

	Sex	BMI	Waist circumference	Hip circumference
Adjusted for age	0.15 (–0.23, 0.52)			
Adjusted for age + BMI	0.32 (–0.10, 0.74)	0.04 (0.00, 0.08)		
Adjusted for age + waist + hip	0.01 (–0.47, 0.48)		0.05 (0.02, 0.08)	–0.03 (–0.07, –0.00)
Adjusted for age + BMI waist + hip	0.01 (–0.45, 0.48)	0.02 (–0.06, 0.09)	0.04 (0.01, 0.08)	–0.04 (–0.07, –0.00)

Data are the unstandardized regression coefficient (95% confidence interval) and represent the unit change in fasting glucose per unit change in the independent variables (BMI unit or waist/hip in cm).

Potential confounders did not change the main results. For example, addition of length of residence to the model that already corrected for age, sex, BMI, waist and hip resulted in an OR of EFG of 1.48 (95% CI 0.62, 3.54), the relative contribution of WC and HC was also unchanged. Thus we did not include these additional variables in the final analysis in order to avoid over fitting.

In addition, we included the waist-to-hip ratio in the analysis rather than hip and waist circumferences individually. The results of the analysis using waist-to-hip ratio did not differ from when waist and hip circumferences were entered as individual variables, i.e. sex difference in EFG were no longer significant. We chose to present the analysis in which waist and hip were entered as independent variables as this is more illustrative of the individual contribution of each of these anthropometric variables.

## Discussion

Sex differences in disease patterns have long been recognized and recently there have been increased calls for differentiating men and women in epidemiological studies in order to promote the development of new insights, and solutions for chronic diseases [34]. This is the first study to explore sex differences in EFG in a West-African migrant population. We obtained evidence to suggest that differences in body fat distribution are related to sex differences in EFG. The results indicated that women were less likely to have EFG than men and that this difference was even greater with correction for generalized obesity (BMI). However, when taking body fat distribution into account, by including both waist and hip circumferences in the model already correcting for BMI, the sex differences in EFG were reduced substantially: with waist circumference being positively and hip circumference being negatively associated with EFG. In further investigation, of fasting glucose as continuous variable, a similar association between the anthropometric variables was observed.

There are some limitations to consider. First, although an elevated fasting glucose is clearly indicative of increased risk of DM [35], up till recently, the classification of appropriate cut-offs to signify that risk has been disputed. Recent consensus has placed the definition of “increased fasting glucose” at glucose >5.5 mmol/L [36]. In this study we used this definition and included participants with known DM. In sensitivity analysis, applying the higher cut-off (FPG ≥6.1 mmol/L), as previously used by the IDF/WHO [16], we found similar results. We also tested the influence of including persons with known diabetes (doctor diagnosed and/or use of medication) on our analysis and found that their exclusion did not influence the associations studied (results not shown).

Second, as our recruitment strategy relied on church attendance, our sample may not adequately represent the Ghanaian population. Evidence suggests that the majority of Ghanaians in Netherlands are involved in faith organizations as these serve both a religious and a social function [4] implying that we are likely to have reasonable representation in our sample. In addition, our strategy succeeded in including non-documented persons, a group that may be expected to be difficult to reach and which can be expected to have differing health outcomes due to differences in health care access. Nonetheless it is not unreasonable to expect that our sample was somewhat biased; persons with suspected health problems may have been more likely to participate.

Third, we did not conduct oral glucose tolerance tests (OGTT), which would have allowed calculation of impaired glucose tolerance (IGT). Gender differences in the prevalence of the two forms of pre-diabetes have been reported, with women being more likely to exhibit impaired glucose tolerance whereas in men, impaired fasting glucose is more common [34], [37]. We cannot comment on the association between body fat distribution and IGT.

Sex differences in EFG could be due to differences in lifestyle, such as smoking, physical activity, alcohol and diet. Sex differences in these variables are well established [38], [39]. We found no significant differences in smoking, physical activity and alcohol consumption in our study population, probably due to the small sample size. Our questionnaire was filled in with the help of ethnically matched interviewers, which may have introduced some bias as participants may have provided socially desirable answers: it is not known if this tendency differs between Ghanaian men and women. Thus we cannot rule out the influence of these important confounders in the observed associations.

Finally, this study was not designed to answer the particular question addressed in this paper. In order to provide insight into the uncertainty of our results we performed a post-hoc power analysis to calculate the power to demonstrate the observed differences in the odds ratio (OR) of EFG among men compared to women. In our study, we found an OR of 1.81 with adjustment for age only; the power to demonstrate such a difference given the number of participants is approximately 45%. Stated otherwise if we were to design this study with 80% power of demonstrating this difference we would have to include approximately 240 persons per sub-group.

Our finding that body fat distribution is associated with fasting glucose, i.e. that a greater hip circumference appears to be negatively associated with an elevated fasting glucose is consistent with studies in other, multi-ethnic populations [22] and with recent reviews [40], [41]. However, it remains puzzling that, while we found no sex differences in the association between fasting glucose and anthropometric measures, when applying a cut-off to define EFG we found a large discrepancy in the odds for EFG between men and women. This could not be explained by differences in the distribution of fasting glucose, or the cut-off used to define EFG (see above). Furthermore, we found no evidence of interaction between sex and the variables included in the analysis.

Our study did not fully explain the sex differences in EFG. Although no longer statistically significant, even when accounting for body fat distribution, women had lower odds of EFG than men. In this study we used anthropometric measurements as a proxy for the assessment of body fat. These measures, when taken together, can adequately represent body fat distribution, with limited intra and inter observer variability. However, they do not discriminate between subcutaneous, visceral and intramuscular fat which are all differently related to DM [42]. Recent studies have shown sex differences in the association between visceral and subcutaneous fat and DM risk. For example there are indications that visceral fat has a stronger association with DM risk among women than among men [43]. In addition, Tulloch-Reid et al found evidence that subcutaneous fat had significantly greater correlations with insulin sensitivity than with visceral fat [44]. Thus, more accurate measures of visceral and subcutaneous fat may shed more light on the sex differences in EFG observed in our study of Ghanaian migrants. Furthermore, we could not account for differences in fat free mass as a potential explanation for the observed differences between men and women. A relatively small hip circumference may reflect a relatively small leg muscle mass [45]. Skeletal muscle is the main target organ for insulin, thus a smaller muscle mass may contribute to higher plasma glucose levels [46].

Furthermore, the relative advantage of women may be due to hormonal influences, as a number of hormones, including sex hormones have been implied in DM risk [47], [48], as well as body fat distribution [49]. It is plausible to expect that changes in hormonal status as a result of menopause would influence women’s likelihood of EFG. Unfortunately we did not have information on menopausal status in our study. We explored this possibility by applying the cut-off age of 50 as a proxy for menopause (30 of the 115 women were older than 50 years). However, we found no differences between the two groups of women in the association between body fat distribution and elevated fasting glucose or in fasting glucose levels in models including all three anthropometric measures.

### Implications

Currently, BMI and WC cut-offs are based on data on European populations due to insufficient evidence for different cut-offs for African populations. However, differences in the association between BMI and WC and disease and mortality risk between white Americans and African Americans have been observed [50]. A recent study by Katzmarzyk et al has indicated that these differences may be sex-specific: they found no differences between African American and white American men in optimal BMI and WC cut-offs whereas in African American women, the optimal BMI and WC values were ∼3 kg/m^2^ and 5 cm higher than in white American women [51].

Considering the paradox in the association between DM and obesity (measured by either BMI or WC) in women, our findings may have implications for the identification of individuals at risk of DM in West African populations. The current emphasis on measuring either BMI or WC [16] based on current cut-offs and without accounting for hip circumference implies that women are more likely to be incorrectly identified as being at risk for DM.

## Conclusion

This is the first study to explore sex differences in the association between body fat distribution and DM in a recent group of West-African migrants. We observed differences in the prevalence of EFG between men and women and found evidence to suggest that despite greater generalized obesity, women’s more favorable fat distribution (characterized by both waist and hip circumference) may confer an advantage. These findings merit replication in a larger population.

## Supporting Information

Table S1 **Correlation between anthropometric measures.**
(DOCX)
