# Supplementary material for: Differences in Body Fat Distribution Play a Role in the Lower Levels of Elevated Fasting Glucose amongst Ghanaian Migrant Women Compared to Men
Source: PLoS One. 2013 Jun 19;8(6):e66516. doi: 10.1371/journal.pone.0066516 (PMC3686715; doi:10.1371/journal.pone.0066516)
Supplement: Table S1 — Correlation between anthropometric measures. (DOCX) [file pone.0066516.s001.docx]

Supplementary material - Differences in body fat distribution may explain the lower levels of elevated fasting glucose in Ghanaian migrant women compared to men.

Table S1: Correlation between anthropometric measures.

|  | overall | Men | Women |
| --- | --- | --- | --- |
| BMI + waist | 0.794 | 0.845 | 0.819 |
| BMI + Hip | 0.786 | 0.753 | 0.759 |
| BMI + WHR | 0.159 | 0.484 | 0.345 |
| Hip + waist | 0.714 | 0.736 | 0.787 |
| WHR + waist | 0.524 | 0.720 | 0.604 |

WHR = Waist-to-Hip ratio; All correlations were highly significant, p≤0.0001
